# Approaches for estimating benefits and costs of interventions in plant biosecurity across invasion phases

**DOI:** 10.1002/eap.2319

**Published:** 2021-05-06

**Authors:** Melissa J. Welsh, James A. Turner, Rebecca S. Epanchin‐Niell, Juan J. Monge, Tarek Soliman, Andrew P. Robinson, John M. Kean, Craig Phillips, Lloyd D. Stringer, Jessica Vereijssen, Andrew M. Liebhold, Tom Kompas, Michael Ormsby, Eckehard G. Brockerhoff

**Affiliations:** ^1^ Scion (NZ Forest Research Institute) P.O. Box 29237 Christchurch 8540 New Zealand; ^2^ Better Border Biosecurity Private Bag 4704 Christchurch 8140 New Zealand; ^3^ AgResearch, Ruakura 10 Bisley Road Hamilton New Zealand; ^4^ Resources for the Future 1616 P Street NW Washington D.C. 20036 USA; ^5^ Market Economics Ltd. Digital Basecamp 1132 Hinemoa Street Rotorua 3010 New Zealand; ^6^ Manaaki Whenua – Landcare Research Private Bag 92170 Auckland 1142 New Zealand; ^7^ Centre of Excellence for Biosecurity Risk Analysis School of BioSciences University of Melbourne Melbourne Victoria 3010 Australia; ^8^ AgResearch Private Bag 4749 Christchurch 8140 New Zealand; ^9^ NZ Institute for Plant and Food Research Private Bag 4704 Christchurch 8140 New Zealand; ^10^ USDA Forest Service Northern Research Station Morgantown West Virginia 26505 USA; ^11^ Faculty of Forestry and Wood Sciences Czech University of Life Sciences Praha 6 – Suchdol CZ 165 21 Czech Republic; ^12^ School of Ecosystem and Forest Sciences University of Melbourne Melbourne Victoria 3010 Australia; ^13^ Ministry for Primary Industries 147 Lambton Quay Wellington 6011 New Zealand; ^14^ Swiss Federal Research Institute WSL Zürcherstrasse 111 Birmensdorf 8903 Switzerland

**Keywords:** benefit–cost analysis, border biosecurity, cost accounting, general equilibrium modeling, invasive alien species, partial equilibrium modeling, phytosanitary measures

## Abstract

Nonnative plant pests cause billions of dollars in damages. It is critical to prevent or reduce these losses by intervening at various stages of the invasion process, including pathway risk management (to prevent pest arrival), surveillance and eradication (to counter establishment), and management of established pests (to limit damages). Quantifying benefits and costs of these interventions is important to justify and prioritize investments and to inform biosecurity policy. However, approaches for these estimations differ in (1) the assumed relationship between supply, demand, and prices, and (2) the ability to assess different types of direct and indirect costs at invasion stages, for a given arrival or establishment probability. Here we review economic approaches available to estimate benefits and costs of biosecurity interventions to inform the appropriate selection of approaches. In doing so, we complement previous studies and reviews on estimates of damages from invasive species by considering the influence of economic and methodological assumptions. Cost accounting is suitable for rapid decisions, specific impacts, and simple methodological assumptions but fails to account for feedbacks, such as market adjustments, and may overestimate long‐term economic impacts. Partial equilibrium models consider changes in consumer and producer surplus due to pest impacts or interventions and can account for feedbacks in affected sectors but require specialized economic models, comprehensive data sets, and estimates of commodity supply and demand curves. More intensive computable general equilibrium models can account for feedbacks across entire economies, including capital and labor, and linkages among these. The two major considerations in choosing an approach are (1) the goals of the analysis (e.g., consideration of a single pest or intervention with a limited range of impacts vs. multiple interventions, pests or sectors), and (2) the resources available for analysis such as knowledge, budget and time.

## Introduction

Globally, invasive nonnative plant pests (including both insects and plant pathogens) cause billions of dollars of damage every year to crops and food stocks, forests and urban trees, and the natural environment (Aukema et al. 2011, Bradshaw et al. 2016). In addition, huge expenses are incurred to prevent or reduce such damages (Pimentel et al. 2005). Understanding and quantifying the economic costs of invasive species is critical to inform optimal investments in all stages of biosecurity, including pathway risk management, surveillance, eradication, and pest management to mitigate damages. Direct impacts of invasive species, such as damage to crops, trees, and plant products, are a major component of their economic impacts, but there are many other impacts to be considered. Indirect impacts include reduced market access for agricultural exports, additional production costs and foregone revenue, as well as losses in non‐market value such as social welfare (Soliman et al. 2012) and ecosystem services (Kovacs et al. 2010). These indirect impacts are often significant and must be considered when assessing the potential benefits of an intervention (Aukema et al. 2011, Leung et al. 2014). The costs of prevention and pest management are also important to consider when estimating the total cost of pest impacts (Turner et al. 2007). Reductions in pest impacts may be achieved by intervening at any stage of the invasion process from pre‐border measures to management of established pests. Pest risk analysis and pathway management prevent pests from entering and establishing in a country (Leung et al. 2014), whereas surveillance is used to detect populations of invasive pests during their establishment phase when cost‐effective eradication and containment measures may still be possible (Epanchin‐Niell et al. 2012). Once an invasive pest is established and widespread, pest management measures mitigate ongoing damages (Goldson et al. 2015).

There are numerous factors to consider when deciding on an effective, economically efficient course of action (Born et al. 2005, Cororaton et al. 2009, Epanchin‐Niell 2017). For example, while eradication efforts can present a large temporary expense, pest management costs may be incurred indefinitely. Furthermore, trade‐offs exist between related activities such as surveillance and eradication and it is necessary to account for these relationships when determining how to allocate resources (Epanchin‐Niell et al. 2012, 2014). While the balance between surveillance and eradication has been explored in some detail, there is much more uncertainty about the relative benefits of investment in prevention (i.e., pre‐border activities like pathway risk management, Leung et al. 2014) vs. investment in measures targeting later stages of invasions such as surveillance and eradication, or long‐term pest management. This is complicated by the fact that successful prevention of pest arrival is difficult to quantify given sparse information on unintentional arrivals of invasive species (Cororaton et al. 2009, Leung et al. 2014). Consequently, prevention measures are not used to their full potential (Cororaton et al. 2009).

This paper aims to provide insights into the methods used to estimate the costs of nonnative plant pests and the value of biosecurity interventions through all the stages of invasion, from pre‐border through post‐border biosecurity to the management of pest spread and damages. Policy makers must make decisions concerning when, where and how much to spend on preventing pests from entering a country vs. managing and mitigating pest damages post‐establishment. These decisions are typically informed by economic models and methods such as cost accounting, partial equilibrium and computable general equilibrium (CGE) modeling (Soliman et al. 2012, Strutt et al. 2013). Cost accounting sums all of the costs directly associated with a pest invasion or biosecurity intervention. Partial equilibrium modeling uses cost accounting as an input, but allows the prices and costs directly associated with a pest or intervention to change due to pest or intervention impacts. CGE models are an economy‐wide extension of partial equilibrium models that incorporate price and cost changes across the whole economy.

While reviews of economic losses due to established invasive species are numerous (Holmes et al. 2009), there are fewer reviews of costs and benefits of biosecurity interventions (Heikkilä 2011, Marbuah et al. 2014). These studies have tended to review specific interventions, such as eradication (Brockerhoff et al. 2010), with fewer reviews across the stages of biosecurity interventions (Olson 2006, Heikkilä 2011, Epanchin‐Niell 2017). They have, however, paid limited attention to comparing the economic methods used to estimate the costs and benefits and particularly the implications of different methods on estimates. Büyüktahtakın and Haight (2018) is one exception, reviewing existing literature on mathematical models applied to optimizing invasive species prevention, surveillance and control. Another is Cororaton et al. (2009), who review literature integrating risk assessment, mitigation, and control costs with economic models for evaluating economic costs and benefits of alternative mitigation and control policies.

Our contribution to the existing reviews is to consider the implications of economic model assumptions on estimates of benefits and costs for interventions across stages of biosecurity. We address which type of approach is most appropriate, given the questions that need to be answered and available resources, and when a quick approach is more appropriate than a more complicated, resource‐demanding model. We evaluate the pros and cons of each approach, the tools and skills required to implement each, and how each has been used historically to assess costs associated with invasive species and the benefits of their management. In doing so we seek to complement previous studies and reviews, considering the influence of the underlying biological assumptions (Born et al. 2005, Binimelis et al. 2007), by considering the underlying economic and methodological assumptions.

The scope of this paper, in terms of pests, impacts, and interventions, is globally relevant and applicable, but presented with a view to applying methods in New Zealand's biosecurity system. We chose New Zealand because it is widely considered to have one of the most comprehensive and best documented biosecurity programs worldwide (Eschen et al. 2015, Goldson et al. 2016). This scope has influenced the selection of examples and studies that are included, especially regarding the gray literature, although the scope of the peer‐reviewed literature consulted is international. The structure of the paper is as follows: We review the types of pest impacts, damages, and costs, and provide an overview of the economic estimation approaches we discuss. In the next section, we describe the methods of our review. The following three sections provide overviews of the main economic approaches, namely cost accounting, partial equilibrium modeling, and CGE modeling (see definitions below). Numerical tools commonly used across the techniques are referred to in these sections. We then cover these tools more systematically and in‐depth, followed by the synthesis and conclusions.

## Types of Impacts, Damages, and Costs

The costs associated with managing pests and establishment of nonnative species can be split into three main categories: (1) prevention costs, (2) surveillance and eradication costs, and (3) costs stemming from impacts and management of established pests. Here, we define prevention costs as those that are incurred by measures used to prevent pests from entering and establishing, including risk assessment and pathway risk management activities. Activities aimed at detecting and eradicating newly established populations are grouped under “surveillance and eradication.” Finally, containment and pest management activities are intended to reduce the spread nonnative pests and to reduce or avoid damages they are causing. Impacts of established pests include direct and indirect damages, and the ongoing management costs incurred by affected industries, farmers, and households.

### Pathway risk management (prevention) costs

Prevention costs are investments in biosecurity measures to prevent pests from entering or establishing in a country. Pathway risk management is the first stage of biosecurity and aims to stop pests being transported to and beyond the border. The impacts and activities at this stage include (1) pest mitigation measures and phytosanitary treatments undertaken in the exporting country (e.g., fumigation, irradiation, heat and cold treatments), (2) border control (customs and biosecurity) costs, (3) phytosanitary treatments applied to imports on arrival (similar to treatments undertaken in the exporting country), and (4) investment in research. The economic costs of these prevention measures may be subsidized by governments but a large part of these costs and associated inconveniences are often borne by the affected industries and can be passed on to consumers. Exporters and importers may pay for inspection and treatment of exported or imported goods, experience delays, or fund research initiatives to improve biosecurity systems. Industries also may incur indirect impacts, which some cost‐assessment approaches account for better than others. Based on an evaluation of risks associated with a particular pathway, importing countries may choose to apply quarantine measures to particular imported commodities including total bans on imports, partial bans (e.g., prohibitions on imports of a commodity produced in a specific region) and phytosanitary treatments. For example, the International Standards on Phytosanitary Measures No. 15 (ISPM 15) is a harmonized phytosanitary treatment for wood packaging materials, specified by the International Plant Protection Convention, which requires all solid wood packaging material in global trade to either be heat treated or fumigated prior to use. Wood pallet producers in the United States reported that conforming to ISPM 15 added an additional US$1.40 cost per pallet (Woodroffe 2010). These costs were incorporated in the Global Trade Analysis model to estimate how activities mandated by ISPM 15 could potentially impact on exports and economic welfare for different countries (Strutt et al. 2013). Studies of wood borers and bark beetle interceptions on wood packaging and pallets at the U.S. border pre‐ and post‐ISPM estimated that these measures resulted in a reduction of arrival by approximately 50% (Haack et al. 2014) and cumulative net benefits exceeding US$11 billion by 2050 (Leung et al. 2014). Peterson et al. (2013) applied a product‐line gravity model to quantify the effects of phytosanitary treatment requirements on fruits and vegetables imported to the USA. They found that while phytosanitary treatment requirements generally reduce imports, these negative impacts tend to decrease (or even disappear) as exporters accumulate experience.

### Surveillance and eradication costs

#### Surveillance

Pathway risk management is never completely effective at excluding new pests from a region, thus post‐border biosecurity measures to detect and eradicate new incursions are necessary. For eradication to be successful, it is best to detect populations while they are small and have a limited spatial extent (Brockerhoff et al. 2010, Tobin et al. 2014). Consequently, it may be necessary to expend considerable resources on surveillance for new incursions. Costs associated with surveillance include expenditure on traps, lures and detection systems, wages for those setting and inspecting traps, getting to and from surveillance sites, as well as diagnostic services and data analysis (Mayo et al. 2003). A number of studies aim to optimize surveillance programs by estimating the density of surveys (e.g., traps) that will provide the lowest combined costs from surveys and detection response (e.g., eradication), while incorporating information about the biology of the pest. These studies consider characteristics such as pest population growth rate. A slow growing population may be easy to eradicate, and thus does not need to be detected quickly, so a low trap density is optimal. A low trap density also may be optimal for a rapidly growing population that will quickly intercept a surveillance trap. However, intermediate growth rates may require a higher trap density to detect a population sufficiently early to intervene (Bogich et al. 2008). Surveillance costs often vary geographically, and it is necessary to account for environmental factors affecting costs. One of the greatest expenses in most surveillance programs is the cost of moving (e.g., driving) among detection points (e.g., trap locations). Blackburn et al. (2017) use routing software to simulate driving times among surveillance grids deployed across various real road networks, then model driving times and total surveillance costs as a function of grid density and road network characteristics.

#### Eradication and containment

A variety of methods are used for eradicating plant pests including host plant removal, pesticide application, and mass trapping (Brockerhoff et al. 2010, Liebhold et al. 2016). Costs include procurement of machinery and traps and operational costs such as labor hours. Blackwood et al. (2012) apply a simple per‐hectare cost derived from historical data to estimate gypsy moth (*Lymantria dispar*) eradication costs. Similarly, Yemshanov et al. (2017) use historical costs for felling and destroying trees to estimate costs for eradicating Asian longhorned beetle (*Anoplophora glabripennis*, ALB) from Toronto, Canada. However, in some cases (e.g., eradication of species for which there is no previous experience or data from eradication programs), data required for cost accounting may not be available (Phillips et al. 2020). In these cases, it may be necessary to extrapolate eradication costs from programs targeting similar species. This approach, used by Brockerhoff et al. (2010), Tobin et al. (2014), and Suckling et al. (2016), uses data from multiple historical eradication programs, such as recorded in the Global Eradication and Response Database (Kean et al. 2019), to regress the cost of eradicating a population from an invaded area. These costs represent the direct operational costs of the eradication programs and do not factor in‐kind costs or costs incurred by people or industries indirectly affected by movement controls or the tools used during the eradication. A prominent containment program is “Slow‐the‐Spread” for gypsy moth in the United States, and there is good information on the benefits and costs of the program (Sharov et al. 2002).

### Impacts of established nonnative species

**Fig. 1:**
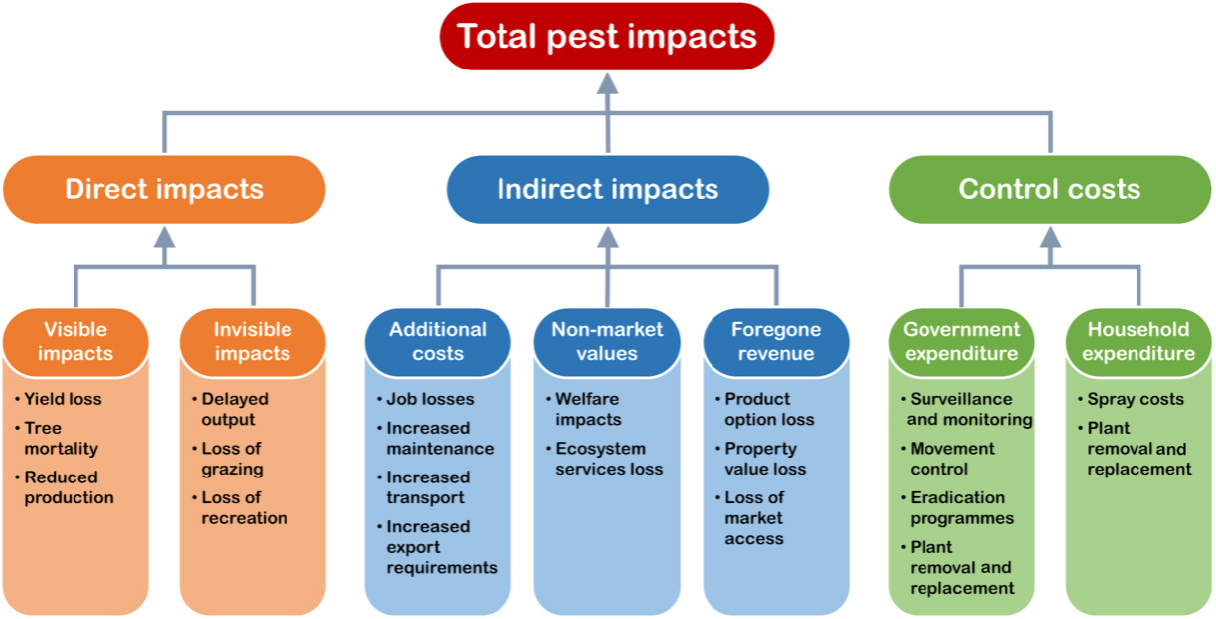
Pest impacts chart, showing examples of various types of impacts a pest can have and how these contribute to the total impact.

Impacts that result from nonnative species establishing a self‐sustaining population in a country where it was previously absent are referred to as impacts of established species. Fig. 1 gives an overview, and examples of the various types of impacts an invading pest might have. Impacts can be direct, such as insects damaging crops, or indirect such as reduced amenity value or market access. These impacts combine to give the total impact of an invasive species, though care must be taken to avoid double counting, as these various impacts may influence each other. For example, pest control costs aim to reduce damages so costs of control and costs of expected damages without control cannot be summed (Holmes et al. 2009).

#### Direct impacts

Examples of direct impacts include yield losses, reduction in crop quality, plant mortality, or delays in harvesting. When pest damages result in lost crops or tree mortality, a direct connection can be made between the pest and these impacts. Some direct impacts may not be immediately noticeable, for example, if an invasive pest reduces the growth rate of trees. Despite some being less visible, direct impacts are relatively easy to quantify and thus are often the main component of impact assessments (Holmes et al. 2009).

#### Indirect impacts

These are secondary effects that result from an invasive species' presence but are not immediately connected. Indirect impacts can include devaluing of resources or outputs, such as reduced property value (e.g., by loss of an attractive specimen tree), lost ecosystem services, damage to market brands, and lost market access for exports, which may in turn have additional downstream effects. Export markets may be harmed due to the establishment of a pest unwanted by trading partners, due either to increased production costs for export (Turner et al. 2007) or even to loss of trading partners in the event of a total ban (Prestemon et al. 2006, Self and Turner 2009). Indirect impacts may also arise from a resurgence of previously controlled pests, for example, the use of pesticides against a new invader impacting beneficial insects such as predators of a pest (Dutcher 2007). In this case, the costs of increased pesticide use can be estimated (Gross and Rosenheim 2011) as well as the loss due to the presence of these previously controlled pests.

Indirect impacts can be categorized into three main types as illustrated in Fig. 1: namely additional costs, non‐market value loss, and foregone revenue. Additional costs are extra spending that occurs in the presence of the pest, such as increased maintenance of machinery or additional treatment of exports necessary to retain market access. Non‐market value losses include losses due to impacts on the health and wellbeing of the people involved and how well they are able to maintain their way of life (Holmes et al. 2009). Non‐market losses also arise from reductions in ecosystem services, such as pollination if pollinators are affected, soil stability relating to erosion and water quality, biodiversity, aesthetic value, regulating services such as climate regulation, maintenance of water quality, and habitat provision, as well as cultural services (Leemans and Groot 2003, Boyd et al. 2013, Vilà and Hulme 2017). Placing a value on these services helps to convey their importance and the role they play in various sectors (Boyd et al. 2013). While non‐market values are a major component of indirect impacts, they are difficult to estimate in monetary terms (Holmes et al. 2009).

#### Control costs

An established invasive pest may trigger a response aimed at reducing damages, by suppressing the pest population or removing host material (Goldson et al. 2015, Barron et al. 2020). Activities include spraying affected areas with pesticides, releasing biological control agents, removing and replacing affected plant material, and monitoring of pest populations to assess control effectiveness. As illustrated in Fig. 1, costs may be incurred by local or national government, landowners, households, or some combination of these. The scale of the control costs depends on the size of the area treated and the intensity of the pest invasion, with large‐scale pest management often coordinated by governing entities or grower cooperatives. Large‐scale control programs may become long‐term eradication efforts such as the 55‐yr program that successfully eradicated the Mediterranean fruit fly, *Ceratitis capitata*, from Bermuda (Hilburn and Dow 1990).

## Selecting Approaches and Literature for Review

### Introduction to approaches to estimating the costs and benefits of interventions

Damage and mitigation costs may be estimated ex ante or ex post, to quantify impacts that are likely to occur or have occurred, respectively. These estimates are increasingly being developed with the aim of informing more efficient or effective biosecurity policy (Epanchin‐Niell 2017). The impacts of invasive pests are varied, with some being more readily quantifiable in economic terms than others. Challenges include limitations posed by a lack of data and uncertainty (Leung et al. 2014), and the diversity of approaches that can be used. In some cases, relatively simple accounting estimates may be sufficient, while for other purposes, a complex general equilibrium model that accounts for changes in supply and demand of goods, and interactions among economic sectors is more appropriate.

The main economic approaches used include cost accounting, partial equilibrium, and CGE modeling. Cost accounting, also referred to as partial budgeting or marginal analysis, is a technique used to quantify all the potential costs of a pest invasion or intervention, in a relatively straightforward, additive manner (Soliman et al. 2010). Partial equilibrium modeling provides an estimate of the change in economic welfare (consumer surplus plus producer surplus) due to, for example, a pest incursion or investment in prevention. The approach employs an economic equilibrium model for a specific supply and demand scenario. CGE models are an “entire economy” extension of partial equilibrium models. Using examples of applications of each approach, we discuss their pros, cons, and potential considerations and biases. Table 1 lists studies that have used each approach to assess the various impacts of pests or prevention measures across the biosecurity spectrum (for an extended list see Data S1: [PaperReview]).

**Table 1:** Representative examples of approaches for estimating costs and benefits of each pest impact type and intervention stage.

Impact or intervention	Cost accounting	Partial equilibrium modeling	Computable general equilibrium modeling
Pathway risk management	Strutt et al. (2013), Haack et al. (2014)	Prestemon et al. (2006), Turner et al. (2007)	Strutt et al. (2013)
Surveillance	Blackburn et al. (2017)		
Eradication and containment	Turner et al. (2004), Bogich et al. (2008), Brockerhoff et al. (2010)		
Control costs	Mayo et al. (2003), Turner et al. (2004)	Epanchin‐Niell et al. (2012), Büyüktahtakın and Haight (2018)	McDermott et al. (2013)
Direct impacts	Aukema et al. (2011), Nghiem et al. (2013), Basse et al. (2015)	Turner et al. (2007), Soliman et al. (2012)	Greer and Saunders (2012), McDermott et al. (2013)
Indirect impacts	Kovacs et al. (2010), Basse et al. (2015)	Turner et al. (2007), Soliman et al. (2012)	Greer and Saunders (2012), McDermott et al. (2013)

Economic approaches differ in scope with respect to the relationships between supply, demand, and prices represented, and linkages among agriculture, forestry and other sectors of the economy. As a result, the ability of different methods to assess all direct and indirect costs also differs. Each economic assessment method requires certain assumptions about the impacted commodity or sector, and how other commodities or sectors may be affected. Cost accounting, for example, assumes fixed prices, with no possibility of substituting affected goods (i.e., the prices of the impacted commodities are fixed so, for example, the possibility that wood processors may decide to use another, lower‐priced timber species is not considered; Turner et al. 2007). CGE modeling does not hold any prices fixed; only how much one product is preferred over another is held constant by assuming a constant elasticity of substitution (e.g., it is assumed wood processors would move to using another species of timber at a constant rate for each percent increase in price; Strutt et al. 2013). This allows the added flexibility, and complication, of product substitution in response to price and availability changes for commodities. Partial equilibrium modeling is a middle ground, allowing price changes in products affected by the pest and its impacts, but keeping the prices of less directly affected products constant. Both cost accounting and partial equilibrium modeling assume a fixed product mix meaning there is no allowance for potential product substitution due to pest impacts and associated price changes. Table 2 gives a summary of assumptions required by each approach.

**Table 2:** General assumptions required by each approach.

Assumptions	Cost accounting	Partial equilibrium modeling	Computable general equilibrium modeling
Impacted commodity prices fixed	Yes	No	No
Fixed product mix (no substitution)	Yes	Yes	No
Other product prices are fixed	NA	Yes	No
Constant elasticity of substitution	NA	NA	Yes

NA, not applicable.

As pests invade, their populations grow and spread over time. To sum all the costs associated with a pest, over an extended period, the value of costs and damages before, during and after the invasion need to be considered. The most accepted method of accounting for changes in relative value over time, all else being held constant, is discounting, which we discuss as part of the numerical toolkit.

### Method of article selection

Many studies have considered the costs and benefits of intervention strategies over one or two invasion stages, using various methods of assessment and optimization. The selection of articles reviewed in this paper involved a formal literature search, while also including gray literature. The literature search was undertaken using a keyword search in Google scholar. Keywords included invasive species, pest, pathway, prevention, search, surveillance, eradication, control, management, cost, and economics. After identifying the approaches to be considered, namely: cost accounting, partial equilibrium modeling, and CGE modeling, papers not using these approaches were excluded. Other approaches, such as input‐output analysis and mathematical modeling methods, are valid and have been used successfully in previous studies. These approaches, however, are used less often or in support of one of the three approaches we discuss. Appendix S1: Table S1 shows the number of papers using each approach to estimate the costs and benefits for each impact type an intervention stage. Many papers assess multiple invasion stages or interventions or use multiple approaches, as such the column and row totals are less than the sum of values in each. The number of times each approach is used in combination with one of the other approaches in shown in Appendix S1: Table S2. The number and percentage of times each approach is used in combination is given at the bottom of the table, again this is not the sum of the column values as some approaches are used in combination with more than one other approach in the same paper. Gray literature was obtained from government departments and research groups, searches in Google scholar, and from secondary citations. Examples in the gray literature were not an exhaustive sample of the literature. This review is not intended to be comprehensive, but rather aims to illustrate the main approaches that have been used across invasive species studies. Each article reviewed was characterized based on the approach taken and the types of costs considered. Specific information was extracted from each article and compiled in a spreadsheet (Data S1: [PaperReview]). Spreadsheet categories used consisted of: Specific pests considered, the countries or locations considered, the number of species, the costs analysed, impacts assessed, the type of analysis used, the discount rate applied, the time horizon considered, and the net present value (NPV) estimated for impacts and/or the benefits of interventions. It was also noted if a sensitivity analysis was included along with any other shortcomings and/or limitations. These included failing to account for interaction among species, such as when scaling up damage estimates from a few highly damaging species, to hundreds of species; omission of discounting despite estimating impacts over an extended time period; and use of very rough estimates for parameter values with little explanation of their derivation or rationale, limiting reproducibility and usability of results. While shortcomings impacted the inclusion of studies as examples in this paper, they must also be addressed in future assessments of invasive species costs.

## Cost Accounting

This was the most common approach used in the literature reviewed (Table 1). Costs can be accrued based on the budget of producers, governments or other entities. For example, values of yield losses or additional control costs are often included when the cost accounting approach is applied to capture the impacts of an invasion on an agricultural producer budget (Turner et al. 2007). Similarly, costs of prevention, surveillance, and research programs are cost items included when assessing impacts on governmental budgets (Epanchin‐Niell et al. 2014). Disentangling expenses incurred by importing vs. exporting countries is crucial when assessing pre‐border costs.

Results from cost accounting can be expressed at the individual producer level or at the regional/national level by aggregating the costs across all producers (Rich et al. 2005). It is possible to summarize costs accrued over time by discounting all future costs to the present value (see below). In general, the cost accounting approach assumes that (1) commodity prices are constant (i.e., insensitive to changes in supply), and (2) there is no change in the mix of products consumed due to invasive species or prevention measures (Turner et al. 2007). Cost accounting estimates also serve as needed input parameters for estimating species impact and prevention costs using other approaches described below.

### Resources needed to use cost accounting

Estimating direct accounting cost is relatively straightforward and typically uses existing data that are frequently recorded by government departments (e.g., biosecurity budget reports), primary producers or other entities. Existing literature may also be used to supplement data and better inform estimates. Application of a cost accounting approach requires information regarding what additional costs will be incurred (e.g., pest damages, control costs, prevention costs), what costs will be reduced (e.g., mitigated damages, eliminated future control costs via eradication), and how returns will be impacted (e.g., reduced outputs). The added costs and reduced returns are weighed against any reduced costs and added returns in a cost–benefit analysis (Rabin et al. 2007). This analysis can easily be implemented in a simple spreadsheet, with minimal economic knowledge required (Soliman et al. 2010).

### Benefits of using cost accounting

Cost accounting is arguably the simplest method for quickly performing approximate cost–benefit assessments, often needed to make rapid decisions around incursion responses. This approach is especially useful in assessing the short‐term impacts of a specific change (Rabin et al. 2007). Immediate action may be needed when an invader is first discovered, despite limited data availability, meaning more finessed approaches are not possible (Baxter and Possingham 2011). Initial decisions about an incursion response are often based on the expected cost of eradication vs. a simple cost accounting estimate for potential damages if the population were to spread unhindered. The expected response cost may simply be compared to the value of the commodity affected, if the ratio is suitably low then eradication may be attempted. For example, when the mite *Bryobia lagodechiana* was detected in a New Zealand rose nursery in 1988, authorities estimated the cost of an eradication attempt at NZ$40,000, while the total annual export value of the flower crop was NZ$400,000 (Baker and Cowley 1989). This overestimates the damage to the crop as it would not likely all be lost, but underestimates the cost of spread to other crops, damage in subsequent years, and additional management costs. The benefit:cost ratio of 10:1 for averted damages to response costs was sufficient to support the decision to attempt eradication. When greater sums are involved, or more time and data are available, more detailed cost accounting can support eradication decisions. For example, MacLeod et al. (2004) assessed the potential costs of Melon thrips (*Thrips palmi*) establishing in the United Kingdom, by estimating the net present value of costs resulting from yield and quality losses, additional research required, plant health certification and loss of exports. They compared this to eradication costs estimated from treating a previous incursion, finding benefit;cost ratios of 4:1 to 110:1. This range exemplifies the considerable uncertainties around the circumstances of current and future incursions (Brown et al. 2019). Cost accounting allows such uncertainties to be treated in a way that is relatively transparent to decision makers (Soliman et al. 2010).

### Drawbacks of using cost accounting

Cost accounting deals well with well‐understood effects, for which impacts can all be quantified directly in monetary terms. However, it fails to account for feedbacks, such as market adjustments and dynamic supply and demand (Soliman et al. 2010). It may overestimate long‐term economic impacts of a pest that damages one commodity if a suitable substitute exists. Issues related to transfers, market adjustments and how expenditures affect the level of prosperity and standards of living among all parties affected (overall welfare) are largely ignored in the cost accounting approach. Furthermore, this approach can only be used to estimate the costs and returns of a specific event or action (Rabin et al. 2007). Because indirect impacts are usually a flow‐on effect of direct impacts, they are typically not considered by a cost accounting approach. Some easier‐to‐quantify impacts such as erosion and carbon sequestration may be included, but a more in‐depth approach is often required to estimate indirect effects such as increased transport and manufacturing costs or wellbeing impacts (Vilà and Hulme 2017).

By considering only the direct effects, cost accounting suggests a robustness that may be misleading if the underpinning economic assumptions do not hold. For example, Surkov et al. (2009) estimated the economic impacts of invasive species to parameterise an optimal allocation model of import inspection. They found that the costs to producers will be overestimated if a cost accounting method was used instead of a partial equilibrium model. Changes in commodity price accounted for in the partial equilibrium model meant that producers could transfer some adverse impacts to domestic and foreign consumers. This will affect the value of import inspections as it depends on estimated costs.

### Examples applying cost accounting to invasive species

Cost accounting is widely applicable to estimating costs associated with invasive species. This approach has been used in many biosecurity benefit–cost analyses, both as part of larger cost estimation projects and as the main assessment method. Costs associated with clover root weevil (*Sitona obsoletus*, CRW) damage in New Zealand, have been estimated by White and Gerard (2006), Basse et al. (2015), and Ferguson et al. (2019). An important direct effect of CRW feeding damage is reduced nitrogen fixation by clover, meaning farmers must replace the lost nitrogen with synthetic fertilizer or accept yield losses. White and Gerard (2006) use a mechanistic whole‐farm model of a single sheep farm in New Zealand to estimate the economic effects of reductions in pasture yield and quality due to CRW. Increased fertilizer use by New Zealand's dairy industry, due to CRW, was estimated by Basse et al. (2015) and Ferguson et al. (2019) using historical urea prices and annual observations of CRW's geographical distribution. The manufacturing of nitrogen fertilizer produces greenhouse gas emissions as a by‐product and applying synthetic nitrogen fertilizer is also a significant source of agricultural emissions of nitrous oxide. Weir and Andrews (2005) estimated the cost of increased emissions due to CRW based on the international price of carbon emissions units. In a medium impact scenario, CRW was estimated to cost NZ$50 million annually in increased emissions from nitrogen fertilizer, and up to NZ$154 million in a high impact scenario. Weir and Andrews (2005) also estimated losses to commercial beekeepers associated with reductions in nectar availability from white clover cover decline.

Emerald ash borer (*Agrilus planipennis*, EAB) has killed millions of ash (*Fraxinus*) trees in North America. Tree mortality necessitates the costly removal of affected trees before they become a hazard. Kovacs et al. (2010) estimated the distribution of ash (*Fraxinus*) trees potentially affected by this pest on developed land in the United States. This is combined with tree removal and replacement cost data, to estimate the direct economic costs of trees lost that can be attributed to EAB. In the case of woodlot owners, the potential value of the timber is lost when a tree dies. One consequence of removing a tree is a reduction in property value. Using house price data, along with the approximate value contribution of a medium sized hardwood tree, Kovacs et al. (2010) also estimated the lost property value associated with EAB invasion.

Nghiem et al. (2013) estimate the economic impact of nonnative invasive species on agricultural systems in Southeast Asia, combining data on production and yield losses with the proportion of pests that are invasive. This provides a simple estimate for value of crop loss attributable to invasive species. Aukema et al. (2011) estimate the economic impact of three high profile pests in the United States. They consider the impacts of each pest on five separate cost categories, spanning government, household and forest landowners. It is assumed that any interactions between these categories are weak and do not warrant consideration. Despite this, the resulting damage costs estimated cannot be summed due to a lack of information regarding the extent of overlap and the risk of double counting.

Cost accounting is the classic approach to estimating surveillance costs. Yemshanov et al. (2019) applied hourly wage levels, average per tree inspection times and total numbers of trees to estimate costs of surveillance for ALB in Toronto, Canada. In the modified cost accounting approach implemented by Mayo et al. (2003), cost records from historical gypsy moth surveillance programs conducted in the eastern United States are used to regress total expenses per trap as a function of environmental variables such as elevation and road density. Data collected from cooperating agencies is collated using a cost accounting approach to capture total operation costs for the “Slow the Spread” pest management program.

## Partial Equilibrium Modeling

**Fig. 2:**
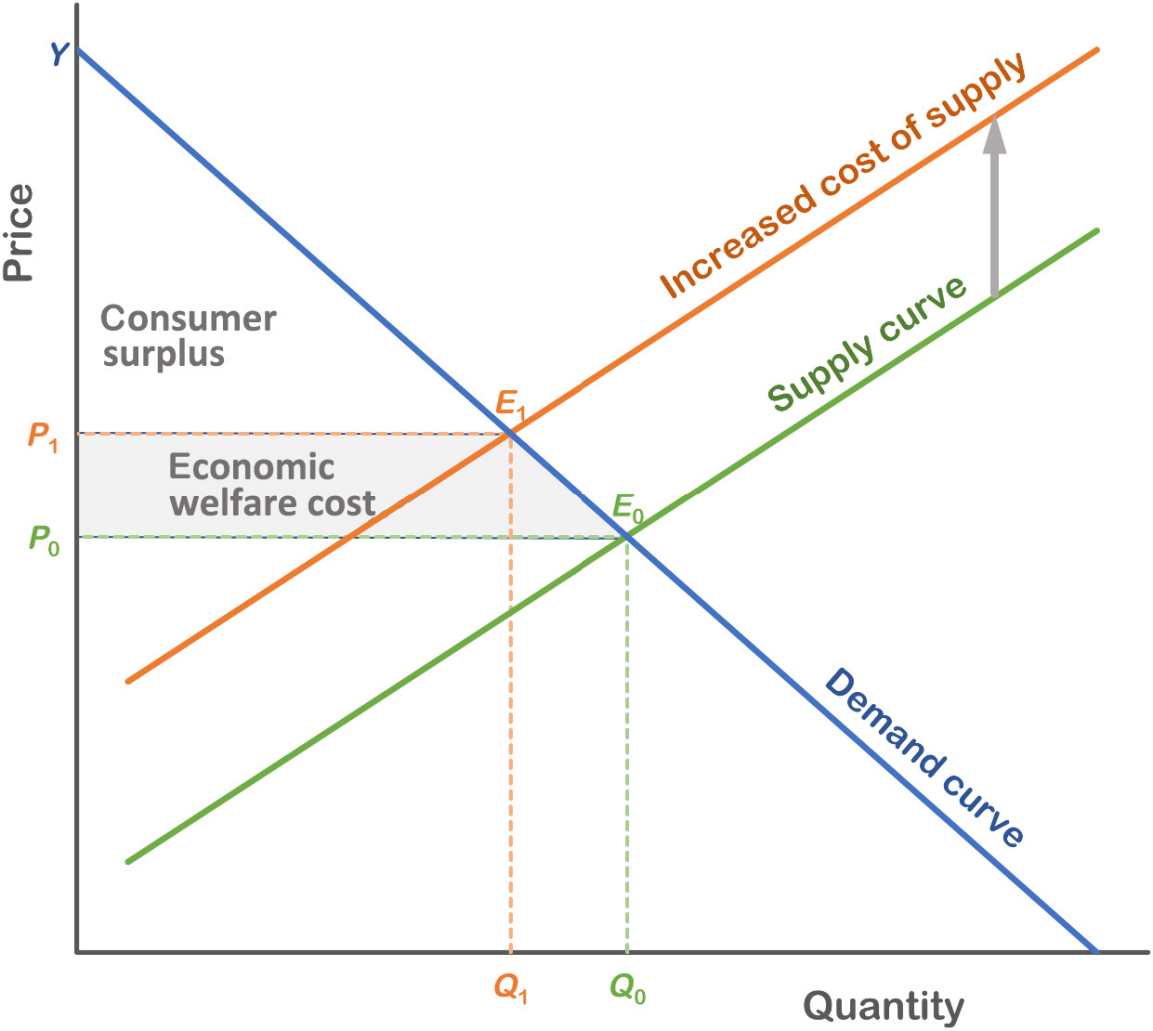
Supply‐demand model, showing price (*P*) and quantity (*Q*) with (_1_) and without (_0_) an increased cost of supply, for example, due to a phytosanitary treatment. The area of the triangle defined by the lines PE, PY, and YE is the consumer surplus of a scenario. The economic welfare cost is the loss in consumer surplus caused by the increased supply cost, meaning the base of the consumer surplus triangle shifts from *P*_0_ *E*_0_ to *P*_1_ *E*_1_.

Partial equilibrium modeling provides an estimate of the change in economic welfare due to a change, e.g., a pest incursion or investment in prevention. In economics, welfare is defined as consumer surplus plus producer surplus. Consumer surplus is the difference between what consumers are willing and able to pay for a good relative to the price of the good. Producer surplus is the difference between the price a producer actually receives for a unit of a good or service, and the minimum price the producer is willing and able to supply for each unit. Fig. 2 shows consumer surplus as the area in the triangle *P*_0_ *E*_0_ *Y* (e.g., *A*_0_ = 1/2(*Y* − *P*_0_)*Q*_0_), where *P*_0_ is the original price, *Q*_0_ is the quantity demanded at that price, *E*_0_ is the equilibrium point (*Q*_0_, *P*_0_), and *Y* is the price at which demand falls to 0. The economic welfare cost of an intervention can be calculated as the change in consumer surplus due to the increased cost of supply. This change in consumer surplus is the area *P*_0_ *E*_0_ *Y* (Fig. 2) minus the area in the triangle *P*_1_ *E*_1_ *Y*.

In assessing the impact of a pest or prevention measure, the partial equilibrium method assumes that: (1) any increased cost of a product due to the pest or its prevention does not influence the cost of other products, and (2) there is no change in the mix of products purchased and consumed as a result of the pest or prevention measures.

Constructing a partial equilibrium model requires first estimating the supply and demand curves for a product of interest (e.g., vehicle imports). Only goods immediately relevant to the scenario under investigation are considered (Jain and Sandhu 2007), but unlike cost accounting, the price of a commodity is not assumed to be fixed. The price of affected goods can change as pest impacts and related activities affect supply and demand. The scope of a partial equilibrium model may vary. Smaller models may involve a single product (e.g., radiata pine logs) or a single market, while large sector models involve multiple product interactions (e.g., forestry and wood products) as well as factors of production (e.g., land, labor, water), in a multi‐market partial equilibrium model. Partial equilibrium models can also be static and thus time invariant, or dynamic allowing for changes over time. Assumptions around market behavior, such as perfect or imperfect competition also need to be made, to accurately attribute changes in supply, demand or prices to the correct influence, i.e., the invasive pest. The rationale behind this “simplified economy” approach lies in pest impacts having limited economic reach. If the invaded area is not large enough to have an economy‐wide effect, impacts are only expected to be realized in the infested commodity and closely related markets.

### Resources needed to use partial equilibrium modeling

Partial equilibrium analysis is often performed using specialized models (e.g., the GFPM; Buongiorno et al. 2003), spreadsheets (e.g., Lincoln Trade and Environment Model; Cagatay and Saunders 2003), or mathematical programming software such as the Advanced Interactive Multidimensional Modeling System (e.g., the Primary Value Chain model; Monge and Wakelin 2019). These models typically rely on comprehensive data sets that include the prices and quantities of each product and country modeled for the base year of the analysis, as well as elasticities (own‐ and cross‐price; e.g., Turner and Buongiorno 2004). Knowledge of how to build the required models, or access to existing models and some knowledge of how they work, is necessary for partial equilibrium analysis. More time may also be required than for cost accounting, to acquire the necessary data, personnel/skills and to put a working model together.

### When to use partial equilibrium modeling?

An advantage offered by partial equilibrium models is that they estimate both market and welfare effects for the sector(s) in question. Partial equilibrium modeling can pick up the effects of feedbacks, such as market adjustments and dynamic supply and demand, which cost accounting cannot. This approach can provide more accurate estimates of actual pest impacts compared to a cost accounting approach when changes in the consumption and/or production volumes are large enough to create possible changes in product prices (Prestemon et al. 2006, Holmes et al. 2009). In contrast to cost accounting, partial equilibrium modeling can account for behavioral responses by producers and consumers as product prices and quantities change due to pest impacts, and thus can estimate changes in producer and consumer welfare (Holmes et al. 2009; Fig. 2). Depending on the extent of the markets included in the analysis, partial equilibrium models could potentially measure the indirect market and economic welfare impacts on sectors, inputs (e.g., labor, chemicals) and natural resources (e.g., land, water) related to the sector(s) in question (Holmes et al. 2009).

### Reasons not to use partial equilibrium modeling

While partial equilibrium models allow for in‐depth analysis of pest impacts, added complexities mean this approach may not be manageable given limited time. Assumptions regarding the direct prevention cost of a phytosanitary policy are needed in order to incorporate the cost into the specific structure of the partial equilibrium model. Partial equilibrium models tend to aggregate products (e.g., radiata pine, Douglas fir, redwood, and eucalyptus logs are aggregated as “industrial roundwood” in the GFPM); thus, if a prevention measure is applied to a specific product (e.g., eucalyptus), the cost of this measure will need to be adjusted based on the product share. (e.g., share of a tree species in total log imports or exports). These assumptions are made on a case‐by‐case basis. To avoid making arbitrary assumptions regarding specific prevention measures for individual species or products in highly aggregated partial equilibrium models, it is advisable to also rely on more detailed impact approaches such as cost accounting (Soliman et al. 2012). It can be difficult to build, use and validate partial equilibrium models and this may prevent analysts from using them more widely. The structure and data inputs of most widely used models, e.g., the GFPM, are well maintained and have been validated and well documented in various peer‐reviewed articles, books and manuals. Without such resources, data requirements may preclude application. Knowledge of the theory behind these models is necessary to be able to interpret results.

### Examples of partial equilibrium modeling

Partial equilibrium modeling has been used by studies as a base from which to estimate costs (such as Aukema et al. 2011), or from which impact values can be extracted for various scenarios (Turner et al. 2007).

Soliman et al. (2012) use partial equilibrium modeling to estimate the change in consumer and producer surplus due to pine wood nematode (*Bursaphelenchus xylophilus*, PWN). The value of forest products in the presence of PWN is compared to the situation where PWN is not established. The net total impact of PWN across the European Union was estimated at €218 million per annum (Soliman et al. 2012).

In cases where some products are used as inputs to the production of other products (e.g., logs used in sawn timber production in the forest products sector), a pathway risk management model may need to be extended to use a multi‐market partial equilibrium model. The GFPM is a dynamic equilibrium model of the world forest products sector, simulating forestry operations across countries and their trade interactions (Buongiorno et al. 2003). The GFPM has been used to estimate the cost of preventing Asian gypsy moth arriving in the United States (Prestemon et al. 2006). A modified version was used by to estimate the impact of the fungal pathogen *Nectria fuckeliana* on forest resources and products in New Zealand. They considered the potential impact of trade bans and increased phytosanitary regulations. Various responses by importers of New Zealand logs are incorporated and compared with the status quo. These studies incorporate multiple products and multiple importing and exporting countries to better estimate the pathway risk management costs associated with a pest in a highly connected market.

## Computable General Equilibrium Modeling

CGE models incorporate all available economic sectors, including the capital and labor sectors and the linkages among these (e.g., labor used in the construction sector to build homes from sawn timber). Prices are not assumed to be fixed but instead depend on elasticity values and the connection between sectors. Consequently, CGE modeling often reflects responses over a long‐time scale. CGE modeling may be static or incorporate time dynamics, and assumptions must be made regarding perfect and imperfect competition and price‐expectations over time. CGE models can use mathematical optimization implemented in specialized software to solve for sector prices and quantities and require comprehensive databases of economic activity by sector (e.g., the Global Trade and Analysis Project [GTAP] database/model; Strutt et al. 2013).

### Resources needed to use computable general equilibrium modeling

Analysts can make use of CGEs through specialist models (e.g., the GTAP model), CGE modeling software (e.g., General Equilibrium Modeling Package), or mathematical programming software (e.g., General Algebraic Modeling System). CGEs rely on social accounting matrices and price and substitution elasticities as model inputs. For national and regional analyses, it is sometimes necessary to downscale national matrices or use specialist software (e.g., Economic Impact Analysis for Planning software in the United States) to obtain regional matrices (Monge et al. 2014).

### When to use computable general equilibrium modeling?

Computable general equilibrium models represent multiple sectors and their inputs and outputs. They can be used to model the economic cost of multiple impacts in different sectors and are helpful for including indirect, or downstream value chain impacts. CGE models allow for prices to change throughout the sectors in the economy in response to shocks and over time. They are useful for estimating an overview of pest impacts into the future. CGE models allow a broader analysis of pest impacts, including more commodities and a greater view of the economy, than both cost accounting and partial equilibrium modeling.

### Reasons to not use computable general equilibrium modeling

Incorporating the whole economy into one model requires a high level of aggregation to be practical. This aggregation makes CGE modeling an excellent tool for high‐level analysis, whereas estimating the impacts on a specific crop such as kiwifruit, may not be possible. Assumptions will need to be made regarding the cost of prevention to incorporate this cost into the specific structure of the CGE model. If the prevention measure is applied to a specific product, its cost will need to be adjusted based on the share of that product in the aggregation. For example, to include the cost of ISPM 15 in the GTAP model an estimate of the share of wood packaging cost in the total product cost was needed (Strutt et al. 2013).

For most pest impact analyses, CGE requires more complexity than is needed for a science‐based cost analysis. Like partial equilibrium models, CGE models are somewhat difficult to build, use and validate, which may preclude analysts from using them more widely.

### Examples of computable general equilibrium modeling

The CGE modeling approach is used in Strutt et al. (2013) to model the economic costs associated with ISPM 15. This prevention measure impacts multiple sectors that use wood packaging, requiring additional inspections of vehicles and fruit imports. Greer and Saunders (2012) estimate the cost of Psa‐V to the kiwifruit industry, 2 yr after it was discovered in New Zealand. This disease was expected to cost the industry between NZ$740 and NZ$885 million in immediate direct costs to net industry returns and delayed costs to the industry's development over 15 yr. Nationally, it was expected that 870–985 jobs would be lost between 2012 and 2016, from sectors that support the kiwifruit industry due to of Psa‐V's effect on production. By 2014 the cost to the industry, in lost exports alone, was already estimated at NZ$930 million (Birnie and Livesey 2014).

McDermott et al. (2013) use a CGE model to contrast the magnitude of simulated damages caused by the invasive emerald ash borer (EAB). The authors argue that most approaches attempting to estimate the cost of invasive species in the literature use fixed‐price approaches, e.g., replacement cost method, and thus reveal a common avoidance of an economic reality, namely that people and markets adjust to biological and economic conditions. They use a CGE model, representing the regional economy of Ohio, USA to demonstrate that endogenous price models generate lower damage estimates (~US$70 million) than exogenous price counterparts (~US$377–$967 million). Endogenous price models allow interaction between markets, households, and production factors through behavioral adjustments to income, output, and substitution effects. McDermott et al. (2013) integrate the annual impacts from EAB through (1) production losses in affected industries, (2) removal and replacement of dead trees by park sectors, (3) removal costs by households in the form of a lower disposable income, and (4) higher government expenditures to remove the dead trees. The authors then estimate different economic equilibria with and without the impacts in the form of different price, quantity, income, and expenditure levels.

## Numerical Toolkit

### Data needs and estimation

Each of the methods outlined above requires input data to parameterise models and provide cost estimates. These input data may be obtained through various combinations of (1) existing data sources, (2) peer‐reviewed and gray literature, (3) *transfer learning* (i.e., the analysis of related data), (4) communication with experts, and (5) optimization approaches.

Existing data may have been collected by the government, industry, or other stakeholders. Care should be taken to understand how these were collected, the quality, and what the data can appropriately be used for. If the data are poor quality or poor fit for the purpose, this must be accounted for by (1) using formal statistical tools such as multiple imputation or weighting, (2) modeling and then inverting the expected flaws in the data, perhaps augmented by data from other sources, or (3) ensuring that concerns about data and plausible impacts are carefully reported and conclusions and recommendations contextualized. This need for quality accounting can mean that decision makers need further support in making decisions that are appropriately conservative to the quality of the data that inform them. There can also be resistance from database managers to use data for any purpose for which it was not collected. Resistance may arise from wariness about the integrity of the research project or due to concerns about data quality, because regulatory data often are collected for transactional as opposed to strategic reasons such that inconsistent standards and missing values are common.

Using acquired data, key relationships can be estimated by a variety of statistical approaches. While a detailed exposition is beyond the scope of this document, a brief description follows. If a preferred functional form has been identified, then parameters are usually estimated with a frequentist approach such as maximum likelihood, or a Bayesian approach. The choice between these two approaches depends on whether other sources of data exist that might usefully be considered, as well as disciplinary traditions and analyst proclivities. A Bayesian framework allows for the relatively easy inclusion of a variety of sources of information and is sometimes easier to deploy than a frequentist framework, especially in complex hierarchical settings (see, for an introduction, Hilborn and Mangel [1997] and McCarthy [2007]). In the absence of a preferred functional form, predictive models may be constructed using smoothing splines, or even machine learning algorithms such as neural nets and random forests. Hastie et al. (2009) provide a readable introduction to the latter. Econometric estimation, such as hedonic analysis, surveys, or travel cost methods may also be used to derive estimates directly from some types of data, often for the estimation of non‐market impacts.

Parameter estimates also may be derived from peer‐reviewed and gray literature directly, or require meta‐analysis or via transfer learning. For example, when estimating supply and demand curves used in equilibrium models, information is needed to specify how the quantity supplied or demanded is likely to change with price. This information can be found in studies such as Michinaka et al. (2011) and Turner and Buongiorno (2004), which provide estimates of demand elasticities for forest product imports. An estimate transfer, using data observed for other pests or in different situations, can be a simple solution if data are scarce or there has been no previous interaction between the pest and environment of interest. This may be a straightforward application of the available data or may require some collation, analysis or manipulation to make the data applicable. A benefit transfer approach is commonly applied when direct analysis is prevented by budget, time or other constraints (Freeman 2014). A meta‐analysis is a synthetic analysis of results or data obtained from existing publications (Ferrer 1998). Bradshaw et al. (2016) compiled a comprehensive database of articles, chapters, and reports estimating the economic cost of invasive insect species around the world to estimate the global annual cost of invasive insect pests. Both approaches require caution, however, because many unknowns come into play when a species is introduced to a new environment.

Parameter estimate solicitation from experts can range from simply asking for an individual's best guess, to engaging multiple experts in a formal expert elicitation process (Burgman 2016, Hemming et al. 2018). The latter takes longer and requires more effort and organization but allows for the validity of estimates to be assessed and improved upon through discussion, resulting in more reliable estimates and better stakeholder engagement. Expert elicitation has been used across a wide range of studies, including for estimation of potential for aquatic invasive species introduction and damages (Rothlisberger et al. 2012, Wittmann et al. 2014). In many of the studies we reviewed, expert opinion was used to estimate parameters needed for the analyses because experimentally measured values were unavailable.

The success of expert elicitation depends on several factors. A larger cohort provides better opportunities for diversity, which, in turn, delivers better results; ideally, at least six (Burgman 2016). There is also the question of what constitutes an expert. The best indicator of an expert's quality is their performance on closely related tasks, often not available at the time of selection (Hemming et al. 2018). Seemingly obvious signs of expertise such as age, seniority, rank, publications, and even peer recommendation, are demonstrably poor indicators of expertise for the purposes of elicitation. The counterintuitive baseline should be, if a person understands the technical content of a question, then their contribution to a pool of experts, via a structured elicitation process, is likely to be valuable.

Mathematical optimization is another tool that may be used to estimate certain parameters. An optimization problem consists of an objective function that is to be minimized or maximized and a set of constraints that define which solutions of the objective function are permissible (Epanchin‐Niell and Hastings 2010, Williams 2013). Mathematical optimization can estimate parameters such as the total expected management costs or producer or consumer welfare effects. For example, Kovacs et al (2010) estimated the expects costs of EAB management by assuming that decision makers would optimally choose among tree removal, herbicide application, tree removal and replacement, or do nothing, and calculating the present value stream of costs accordingly. Similarly, CGE and partial equilibrium models can use mathematical optimization to solve for the product or sector prices and quantities that maximize consumer and producer surplus for all products involved (Yates and Rehman 2002, Buongiorno et al. 2003, Strutt et al. 2013).

### Aggregating data and results

The benefits of implementing a biosecurity measure are realized in future costs and damages that are mitigated or prevented. These impacts may involve multiple pests and occur in multiple sectors over an extended period. Fumigation of a container will remove most species, not just the target. Looking for one species may lead inspectors to find others of interest. The costs of adding species to a “watchlist” will thus depend on what species are already on it. In the context of a cost‐benefit analysis, the spill over costs and benefits of intervening need to be considered. In assessing the damages caused by an invasive pest, value estimates may be available for several aspects of the economy. These seemingly separate areas of impact may interact with each other or share common damages, which prevents aggregation of their estimates. For example, Aukema et al. (2011) estimate the cost of invasive species to federal and local government in the United States, as well as to residential property prices, households, and the timber industry. These cost categories are unable to be summed due to risk of double counting the impacts. For example, transfers between local and federal governments mean there is potential overlap in their expenditures, and residential property values may overlap with household expenditures. More detailed data are required prior to aggregation to avoid risk of risk of double counting. Analogously, pests that occupy similar habitats or depend on similar resources interact with one another and influence each other such that their damages cannot be aggregated as a simple sum.

Another aspect of aggregation is summing costs as a species spreads across the landscape. A pest generally enters a new environment as a small population, in a single location. which then spreads and grows over time. To meaningfully quantify pest impacts, estimates should account for how and when specific regions and industries are likely to be affected. These factors influence the cost efficiency of containment and eradication attempts, as well as where surveillance may best be placed (Sharov and Liebhold 1998, Epanchin‐Niell et al. 2014). In addition, analyses must account for changes in the value of currency and costs of intervention measures over time (Epanchin‐Niell and Liebhold 2015). The most widely used method for this is discounting, which is discussed further in the section below.

Adding together multiple uncertain estimates can compound errors. As such, sensitivity analysis is needed to assess how changes or uncertainty in individual parameters affect the overall outcome. Most studies discussed in this manuscript undertake a sensitivity analysis of some kind, varying in their complexity and output information. Harris Consulting (2003) simply estimate the outcome using a few different values of each parameter and show how much the results differ for the best estimates used in the main analysis. Nghiem et al. (2013) and Basse et al. (2015) conduct more sophisticated sensitivity analyses, using Monte Carlo simulations to construct probability distributions of possible outcomes.

Often results from models require further manipulation before they can be used in a decision context. For example, a model may predict the diameter growth of invasive trees, whereas the basal area per hectare of the invasive population is the measure of interest. While it could be tempting to convert the average diameter per hectare to an average basal area, the estimate would be biased. Jensen's Inequality states that the mean of a convex (concave) function of a random variable is always higher (lower) than the same function of the mean of the random variable (Jensen 1906). The tree‐level basal area should be computed before the results are summed or averaged. As a rule of thumb, analysts should always try to *aggregate late*.

### Discounting

Discounting of costs is often incorporated allow for the aggregation or comparison of costs and benefits over time. A dollar is worth more today than it would be worth next year, due to the opportunity to earn interest on the money received today (Harrison 2010). The discount rate represents the return an investor would expect to receive on a similar investment of equal risk and signifies how much we care about the future compared to today (MacIntyre and Hellstrom 2015).

Various discount rates and approaches to discounting have been argued (Young 2002, Parker 2011). Standard exponential discounting reduces the value of the future dollar by a constant proportion over time. Appendix S1: Fig. S1 shows how two different exponential discount rates influence projected cost, with a higher discount rate placing more value on today, than a lower discount rate. Time has less effect on values when the applied discount rate is lower. Hyperbolic discounting changes the discount rate over time, with even greater weight placed on costs and impacts now compared with the near future (Appendix S1: Fig. S2). The proportional difference in values diminishes over time, such that the difference between two distant futures becomes negligible (Rubinstein 2003). There has been considerable controversy over how discounting should be applied to long‐horizon environmental issues such as climate change and biosecurity (Gollier and Hammitt 2014). Because there is no current consensus, economists must be particularly clear and explicit about what discounting assumptions they are using, and ideally should demonstrate the effects of these assumptions on their results and conclusions.

## Synthesis and Conclusions

**Fig. 3:**
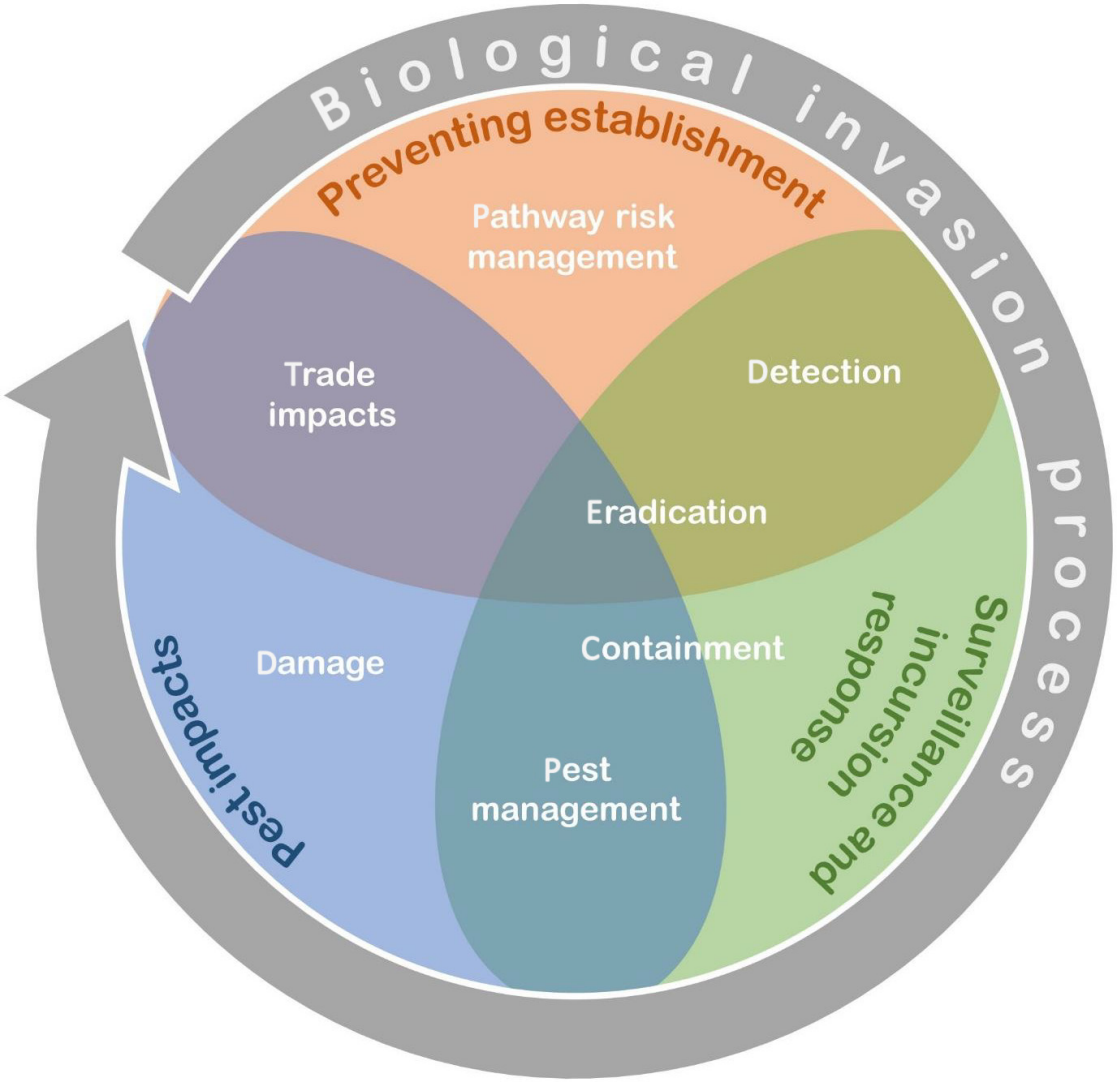
Biosecurity interventions and impacts (white text) interact in potentially complex ways at different stages of the invasion process, from preventing establishment through post‐border surveillance and response to the impacts of established pests, pathogens and weeds. Economic analyses of pest invasions need to consider these factors and their interactions.

Each stage of the invasion process and associated biosecurity interventions interact with each other. These interactions should be considered when analyzing the costs and benefits of managing and targeting invasions. Fig. 3 illustrates how each of the main stages interact with each other, where the overlaps lie and the general direction of influence. Having better surveillance in place increases the chance of eradication efforts succeeding. In turn, successful eradication reduces the need for long‐term management and prevents a pest from doing further damage. Trade impacts come from multiple directions; measures to prevent pest entry make trade more difficult, but so do establishments of pests that induce market access restrictions. Effective pathway risk management reduces pest arrival rates, reducing the need to eradicate, or otherwise manage a pest. The need for surveillance, however, cannot be eliminated. Understanding these balances in biosecurity is important. To account for all of these interactions, a sophisticated analytic approach is needed. The level of sophistication required will depend on the pests considered, the tools available to manage establishment and damages, and the resources available for the analysis.

We have described and discussed several economic approaches, of differing complexity and output accuracy, for estimating the impacts and costs associated with invasive species. Each approach has its benefits, as well as reasons why it may not be selected. Given the discussion and examples above, how can an analyst or researcher decide which approach is best for their application? There is no “one size fits all” method for selecting an analytic approach. However, two major considerations are the goals of the analysis and the context of the decision making. Analysts need to consider what types of insights are desired and the knowledge, budget, and time available. In terms of goals, the intended use of the results, and hence the required analytic outputs, should help guide selection. For example, does the focal decision concern a single pest and a single biosecurity measure, with perhaps a limited range of potential impacts, or is the focus on multiple biosecurity decisions affecting multiple pests or multiple industries and products?

In the case of deciding what amount to invest in a single intervention measure, for a single pest, cost accounting may be the best approach. Partial equilibrium modeling may provide a more accurate picture, incorporating a wider sphere of impacts and costs, but the extra detail and complexity may be unnecessary when addressing impacts over a limited time frame. More complex decisions, involving multiple industries, pests, or interventions (e.g., allocating resource among prevention and surveillance activities) may be informed better by a partial equilibrium model in comparison with cost accounting. When faced with limited time or data resources for analysis a cost accounting approach may be necessary to inform decision makers in time. Similarly, a CGE model allows for a comprehensive analysis of pest impacts on the whole economy, but a simpler approach may be preferred if impacts are anticipated to be within a single small sector of the economy. In some cases, the level of aggregation generally required for implementing more complex CGE models, may obscure important details. For example, a pest may cause large damages in the cropping sector but affect only a subset of crops. Without sufficient detail in the model, it can be difficult to know which crops would benefit most from intervention. It also pays to remember, that no matter how detailed and complex a model is, it will always be wrong. The approaches described are useful tools for estimating what is likely to happen, but can never fully represent all of the nuances of reality (Box and Draper 1987). Even so, some models are less wrong and can be validated based on theory or data. According to McCarl and Apland (1986), there are two types of validation approaches that may be applied to advanced economic models (e.g., PE and CGE): validation by construct and validation by results. Validation by results systematically compares the results of the model against historical observations, outputs of other similar models, or with subject‐matter experts. Validation by construct, however, ensures that model structure is consistent with economic theory and includes sound assumptions (Buongiorno et al. 2003, Dixon and Rimmer 2013).

Generally, it is best to start simple and then incorporate only as much detail and complexity as is needed to answer the questions being asked. As the impacts of the species and available prevention and mitigation measures become better understood, simple models can be extended.

## Supporting information

Appendix S1

Data S1

## References

[eap2319-bib-0001] Aukema, J. E., et al. 2011. Economic impacts of non‐native forest insects in the continental United States. PLoS ONE 6:e24587. 21931766 10.1371/journal.pone.0024587 PMC3170362

[eap2319-bib-0002] Baker, R., and J. Cowley. 1989. Evaluation of the economic impact of newly introduced pests. New Zealand Journal of Forestry Science 19:330–334.

[eap2319-bib-0003] Barron, M. C., A. M. Liebhold, J. M. Kean, B. Richardson, and E. G. Brockerhoff. 2020. Habitat fragmentation and eradication of invading insect herbivores. Journal of Applied Ecology 57:590–598.

[eap2319-bib-0004] Basse, B., C. B. Phillips, S. Hardwick, and J. M. Kean. 2015. Economic benefits of biological control of *Sitona* *obsoletus* (clover root weevil) in Southland pasture. New Zealand Plant Protection 68:218–226.

[eap2319-bib-0005] Baxter, P. W., and H. P. Possingham. 2011. Optimizing search strategies for invasive pests: learn before you leap. Journal of Applied Ecology 48:86–95.

[eap2319-bib-0006] Binimelis, R., W. Born, I. Monterroso, and B. Rodríguez‐Labajos. 2007. Socio‐economic impact and assessment of biological invasions. Pages 331–347 *in* W. Nentwig, editor. Biological invasions. Springer, Berlin, Germany.

[eap2319-bib-0007] Birnie, D., and A. Livesey. 2014. Lessons learned from the response to Psa‐V. Sapere Research Group Limited, Wellington, New Zealand.

[eap2319-bib-0008] Blackburn, L., R. Epanchin‐Niell, A. Thompson, and A. Liebhold. 2017. Predicting costs of alien species surveillance across varying transportation networks. Journal of Applied Ecology 54:225–233.

[eap2319-bib-0009] Blackwood, J. C., L. Berec, T. Yamanaka, R. S. Epanchin‐Niell, A. Hastings, and A. M. Liebhold. 2012. Bioeconomic synergy between tactics for insect eradication in the presence of Allee effects. Proceedings of the Royal Society B 279:2807–2815. 22438497 10.1098/rspb.2012.0255 PMC3367787

[eap2319-bib-0010] Bogich, T. L., A. M. Liebhold, and K. Shea. 2008. To sample or eradicate? A cost minimization model for monitoring and managing an invasive species. Journal of Applied Ecology 45:1134–1142.

[eap2319-bib-0011] Born, W., F. Rauschmayer, and I. Bräuer. 2005. Economic evaluation of biological invasions—a survey. Ecological Economics 55:321–336.

[eap2319-bib-0012] Box, G. E. P., and N. R. Draper. 1987. Empirical model‐building and response surfaces. John Wiley & Sons, Oxford, UK.

[eap2319-bib-0013] Boyd, I., P. Freer‐Smith, C. Gilligan, and H. Godfray. 2013. The consequence of tree pests and diseases for ecosystem services. Science 342:1235773. 24233727 10.1126/science.1235773

[eap2319-bib-0014] Bradshaw, C. J. A., B. Leroy, C. Bellard, D. Roiz, C. Albert, A. Fournier, M. Barbet‐Massin, J.‐M. Salles, F. Simard, and F. Courchamp. 2016. Massive yet grossly underestimated global costs of invasive insects. Nature Communications 7:12986. 10.1038/ncomms12986 PMC5059451 27698460

[eap2319-bib-0015] Brockerhoff, E. G., A. M. Liebhold, B. Richardson, and D. M. Suckling. 2010. Eradication of invasive forest insects: concepts, methods, costs and benefits. New Zealand Journal of Forestry Science. 40:S117–S135.

[eap2319-bib-0016] Brown, K., C. Phillips, K. Broome, C. Green, R. Toft, and G. Walker. 2019. Feasibility of eradicating the large white butterfly (Pieris brassicae) from New Zealand: data gathering to inform decisions about the feasibility of eradication. Pages 364–369 *in* C. R. Veitch, M. N. Clout, A. R. Martin, J. C. Russell, and C. J. West, editors. Island invasives: Scaling up to meet the challenge. Occasional paper SSC. Vol 62. IUCN, Gland, Switzerland.

[eap2319-bib-0017] Buongiorno, J., S. Zhu, D. Zhang, J. Turner, and D. Tomberlin. 2003. The global forest products model: structure, estimation, and applications. Elsevier, Amsterdam, The Netherlands.

[eap2319-bib-0018] Burgman, M. A. 2016. Trusting judgements: How to get the best out of experts. Cambridge University Press, Cambridge, UK.

[eap2319-bib-0019] Büyüktahtakın, İ. E., and R. G. Haight. 2018. A review of operations research models in invasive species management: state of the art, challenges, and future directions. Annals of Operations Research 271:357–403.

[eap2319-bib-0020] Cagatay, S., and C. M. Saunders. 2003. Lincoln Trade and Environment Model (LTEM): an agricultural multi‐country, multi‐commodity partial equilibrium framework. Research Report. Lincoln University, Agribusiness and Economics Research Unit, Lincoln, Canterbury, New Zealand.

[eap2319-bib-0021] Cororaton, C. B., D. Orden, and E. Peterson. 2009. A review of literature on the economics of invasive species. Global issues initiative working paper, global issues initiative. Virginia Tech. Global Issues Initiative, Blacksburg, Virginia, USA.

[eap2319-bib-0022] Dixon, P. B., and M. T. Rimmer. 2013. Validation in computable general equilibrium modeling. Pages 1271–1330 *in* P. B. Dixon and D. W. Jorgensen, editors. Handbook of computable general equilibrium modelling. Volume 1. Elsevier, Amsterdam, The Netherlands.

[eap2319-bib-0023] Dutcher, J. D. 2007. A review of resurgence and replacement causing pest outbreaks in IPM. Pages 27–43 *in* A. Ciancio and K. G. Mukerji, editors. General concepts in integrated pest and disease management. Springer, Dordrecht, The Netherlands.

[eap2319-bib-0024] Epanchin‐Niell, R. S. 2017. Economics of invasive species policy and management. Biological Invasions 19:3333–3354.

[eap2319-bib-0025] Epanchin‐Niell, R. S., E. G. Brockerhoff, J. M. Kean, and J. A. Turner. 2014. Designing cost‐efficient surveillance for early detection and control of multiple biological invaders. Ecological Applications 24:1258–1274. 29160647 10.1890/13-1331.1

[eap2319-bib-0026] Epanchin‐Niell, R. S., R. G. Haight, L. Berec, J. M. Kean, and A. M. Liebhold. 2012. Optimal surveillance and eradication of invasive species in heterogeneous landscapes. Ecology Letters 15:803–812. 22642613 10.1111/j.1461-0248.2012.01800.x

[eap2319-bib-0027] Epanchin‐Niell, R. S., and A. Hastings. 2010. Controlling established invaders: integrating economics and spread dynamics to determine optimal management. Ecology Letters 13:528–541. 20455926 10.1111/j.1461-0248.2010.01440.x

[eap2319-bib-0028] Epanchin‐Niell, R. S., and A. M. Liebhold. 2015. Benefits of invasion prevention: effect of time lags, spread rates, and damage persistence. Ecological Economics 116:146–153.

[eap2319-bib-0029] Eschen, R., K. Britton, E. Brockerhoff, T. Burgess, V. Dalley, R. Epanchin‐Niell, K. Gupta, G. Hardy, Y. Huang, and M. Kenis. 2015. International variation in phytosanitary legislation and regulations governing importation of plants for planting. Environmental Science & Policy 51:228–237.

[eap2319-bib-0030] Ferguson, C. M., et al. 2019. Quantifying the economic cost of invertebrate pests to New Zealand's pastoral industry. New Zealand Journal of Agricultural Research 62:255–315.

[eap2319-bib-0031] Ferrer, R. L.. 1998. Graphical methods for detecting bias in meta‐analysis. Family Medicine‐Kansas City 30:579–583. 9773289

[eap2319-bib-0032] Freeman III, A. M., J. A. Herriges, and C. L. Kling. 2014. The measurement of environmental and resource values: theory and methods. Third edition. RFF Press, Routledge, New York.

[eap2319-bib-0033] Goldson, S. L., B. I. P. Barratt, and K. F. Armstrong. 2016. Invertebrate biosecurity challenges in high‐productivity grassland: The New Zealand example. Frontiers in Plant Science 7:1670. 27895651 10.3389/fpls.2016.01670 PMC5108919

[eap2319-bib-0034] Goldson, S. L., G. W. Bourdôt, E. G. Brockerhoff, A. E. Byrom, M. N. Clout, M. S. McGlone, W. A. Nelson, A. J. Popay, D. M. Suckling, and M. D. Templeton. 2015. New Zealand pest management: current and future challenges. Journal of the Royal Society of New Zealand 45:31–58.

[eap2319-bib-0035] Gollier, C., and J. K. Hammitt. 2014. The long‐run discount rate controversy. Annual Review of Resource Economics. 6:273–295.

[eap2319-bib-0036] Greer, G., and C. M. Saunders. 2012. The costs of Psa‐V to the New Zealand kiwifruit industry and the wider community. Commissioned report. Lincoln University, Agricultural Economics Research Unit, Lincoln, New Zealand.

[eap2319-bib-0037] Gross, K., and J. A. Rosenheim. 2011. Quantifying secondary pest outbreaks in cotton and their monetary cost with causal‐inference statistics. Ecological Applications 21:2770–2780. 22073658 10.1890/11-0118.1

[eap2319-bib-0038] Haack, R. A., K. O. Britton, E. G. Brockerhoff, J. F. Cavey, L. J. Garrett, M. Kimberley, F. Lowenstein, A. Nuding, L. J. Olson, and J. Turner. 2014. Effectiveness of the International Phytosanitary Standard ISPM No. 15 on reducing wood borer infestation rates in wood packaging material entering the United States. PLoS ONE 9:e96611. 24827724 10.1371/journal.pone.0096611 PMC4020780

[eap2319-bib-0039] Harris Consulting . 2003. Asian gypsy moth: assessment of potential economic impacts. Report Prepared for MAF Policy.

[eap2319-bib-0040] Harrison, M. 2010. Valuing the future: the social discount rate in cost‐benefit analysis. Visiting researcher paper. Productivity Commission, Canberra, Australian Capital Territory, Australia.

[eap2319-bib-0041] Hastie, T., R. Tibshirani, and J. Friedman. 2009. The elements of statistical learning: data mining, inference, and prediction. Second edition. Springer‐Verlag, New York, New York, USA.

[eap2319-bib-0042] Heikkilä, J. 2011. Economics of biosecurity across levels of decision‐making: A review. Agronomy for Sustainable Development 31:119–138.

[eap2319-bib-0043] Hemming, V., M. A. Burgman, A. M. Hanea, M. F. McBride, and B. C. Wintle. 2018. A practical guide to structured expert elicitation using the IDEA protocol. Methods in Ecology and Evolution 9:169–180.

[eap2319-bib-0044] Hilborn, R., and M. Mangel. 1997. The ecological detective: confronting models with data. Princeton University Press, Princeton, New Jersey, USA.

[eap2319-bib-0045] Hilburn, D. J., and R. L. Dow. 1990. Mediterranean fruit fly, Ceratitis capitata, eradicated from Bermuda. Florida Entomologist 73:342–343.

[eap2319-bib-0046] Holmes, T. P., J. E. Aukema, B. Von Holle, A. Liebhold, and E. Sills. 2009. Economic impacts of invasive species in forest past, present, and future. Ecology and Conservation Biology, 2009. Annals of the New York Academy of Sciences 1162:18–38. 19432643 10.1111/j.1749-6632.2009.04446.x

[eap2319-bib-0047] Jain, T. R. 2007. Quantitative methods. V.K. Publications, Daryaganj, New Delhi, India.

[eap2319-bib-0048] Jensen, J. L. W. V. 1906. Sur les fonctions convexes et les inégalités entre les valeurs moyennes. Acta Mathematica 30:175–193.

[eap2319-bib-0049] Kean, J., et al. 2019. Global eradication and response database. http://b3.net.nz/gerda

[eap2319-bib-0050] Kovacs, K. F., R. G. Haight, D. G. McCullough, R. J. Mercader, N. W. Siegert, and A. M. Liebhold. 2010. Cost of potential emerald ash borer damage in U.S. communities, 2009–2019. Ecological Economics 69:569–578.

[eap2319-bib-0051] Leemans, R., and R. S. de Groot. 2003. Millennium ecosystem assessment: ecosystems and human well‐being: a framework for assessment. Island Press, Washington, D.C., USA.

[eap2319-bib-0052] Leung, B., M. R. Springborn, J. A. Turner, and E. G. Brockerhoff. 2014. Pathway‐level risk analysis: the net present value of an invasive species policy in the US. Frontiers in Ecology and the Environment 12:273–279.

[eap2319-bib-0053] Liebhold, A. M., L. Berec, E. G. Brockerhoff, R. S. Epanchin‐Niell, A. Hastings, D. A. Herms, J. M. Kean, D. G. McCullough, D. M. Suckling, and P. C. Tobin. 2016. Eradication of invading insect populations: from concepts to applications. Annual Review of Entomology 61:335–352. 10.1146/annurev-ento-010715-023809 26667377

[eap2319-bib-0054] MacIntyre, P., and J. Hellstrom. 2015. An evaluation of the costs of pest wasps (*Vespula* species) in New Zealand. International Pest Control 57:162.

[eap2319-bib-0055] MacLeod, A., J. Head, and A. Gaunt. 2004. An assessment of the potential economic impact of *Thrips* *palmi* on horticulture in England and the significance of a successful eradication campaign. Crop Protection 23:601–610.

[eap2319-bib-0056] Marbuah, G., I.‐M. Gren, and B. McKie. 2014. Economics of harmful invasive species: A review. Diversity 6:500–523.

[eap2319-bib-0057] Mayo, J. H., T. J. Straka, and D. S. Leonard. 2003. The cost of slowing the spread of the gypsy moth (Lepidoptera: Lymantriidae). Journal of Economic Entomology 96:1448–1454. 14650517 10.1603/0022-0493-96.5.1448

[eap2319-bib-0058] McCarl, B. A., and J. Apland. 1986. Validation of linear programming models. Southern Journal of Agricultural Economics 18:55–164.

[eap2319-bib-0059] McCarthy, M. A. 2007. Bayesian methods for ecology. Cambridge University Press, Cambridge, UK.

[eap2319-bib-0060] McDermott, S. M., D. C. Finnoff, and J. F. Shogren. 2013. The welfare impacts of an invasive species: endogenous vs. exogenous price models. Ecological Economics 85:43–49.

[eap2319-bib-0061] Michinaka, T., S. Tachibana, and J. A. Turner. 2011. Estimating price and income elasticities of demand for forest products: cluster analysis used as a tool in grouping. Forest Policy and Economics 13:435–445.

[eap2319-bib-0062] Monge, J. J., H. L. Bryant, and D. P. Anderson. 2014. Development of regional social accounting matrices with detailed agricultural land rent data and improved value‐added components for the USA. Economic Systems Research 26:486–510.

[eap2319-bib-0063] Monge, J. J., and S. J. Wakelin. 2019. Geographically‐explicit, dynamic partial equilibrium model of regional primary value chains—mathematical formulation and application to forestry in the Northland region of New Zealand. Computers and Electronics in Agriculture 156:145–158.

[eap2319-bib-0064] Nghiem, L. T. P., T. Soliman, D. C. J. Yeo, H. T. W. Tan, T. A. Evans, J. D. Mumford, R. P. Keller, R. H. A. Baker, R. T. Corlett, and L. R. Carrasco. 2013. Economic and environmental impacts of harmful non‐indigenous species in Southeast Asia. PLoS ONE 8:e71255. 23951120 10.1371/journal.pone.0071255 PMC3739798

[eap2319-bib-0065] Olson, L. J. 2006. The economics of terrestrial invasive species: A review of the literature. Agricultural and Resource Economics Review 35:178–194.

[eap2319-bib-0066] Parker, C. 2011. Economics like there's no tomorrow. New Zealand Institute of Economic Research (NZIER), Wellington, New Zealand.

[eap2319-bib-0067] Peterson, E., J. Grant, D. Roberts, and V. Karov. 2013. Evaluating the trade restrictiveness of phytosanitary measures on U.S. fresh fruit and vegetable imports. American Journal of Agricultural Economics 95:842–858.

[eap2319-bib-0068] Phillips, C. B., K. Brown, C. Green, R. Toft, G. Walker, and K. Broome. 2020. Eradicating the large white butterfly from New Zealand eliminates a threat to endemic Brassicaceae. PLoS ONE 15:e0236791. 32760094 10.1371/journal.pone.0236791 PMC7410255

[eap2319-bib-0069] Pimentel, D., R. Zuniga, and D. Morrison. 2005. Update on the environmental and economic costs associated with alien‐invasive species in the United States. Ecological Economics 52:273–288.

[eap2319-bib-0070] Prestemon, J. P., S. Zhu, J. A. Turner, J. Buongiorno, and R. Li. 2006. Forest product trade impacts of an invasive species: Modelling structure and intervention trade‐offs. Agricultural and Resource Economics Review 35:128–143.

[eap2319-bib-0071] Rabin, J., C. McGarrity, and M. Banasiak. 2007. Partial budgeting: A financial management tool. USDA, Northeast Region, Sustainable Agriculture for Research & Education (SARE) in Cooperation with Rutgers Cooperative Extension, New Brunswick, New Jersey, USA.

[eap2319-bib-0072] Rich, K., G. Miller, and A. Winter‐Nelson. 2005. A review of economic tools for the assessment of animal disease outbreaks. Revue Scientifique et Technique‐Office International des Epizooties 24:833. 16642754

[eap2319-bib-0073] Rothlisberger, J. D., D. C. Finnoff, R. M. Cooke, and D. M. Lodge. 2012. Ship‐borne nonindigenous species diminish Great Lakes ecosystem services. Ecosystems 15:1–15.

[eap2319-bib-0074] Rubinstein, A. 2003. “Economics and psychology”? The case of hyperbolic discounting. International Economic Review 44:1207–1216.

[eap2319-bib-0075] Self, N. M., and J. A. Turner. 2009. Market access for New Zealand forest products: An economic and environmental case for development of alternative phytosanitary treatments. New Zealand Journal of Forestry Science 39:13.

[eap2319-bib-0076] Sharov, A. A., D. Leonard, A. M. Liebhold, E. A. Roberts, and W. Dickerson. 2002. “Slow the spread”: A national program to contain the gypsy moth. Journal of Forestry 100:30–36.

[eap2319-bib-0077] Sharov, A. A., and A. M. Liebhold. 1998. Bioeconomics of managing the spread of exotic pest species with barrier zones. Ecological Applications 8:833–845. 10.1111/j.0272-4332.2004.00486.x 15357807

[eap2319-bib-0078] Soliman, T., M. C. M. Mourits, A. G. J. M. Oude Lansink, and W. van der Werf. 2010. Economic impact assessment in pest risk analysis. Crop Protection 29:517–524.

[eap2319-bib-0079] Soliman, T., M. C. M. Mourits, W. van der Werf, G. M. Hengeveld, C. Robinet, and A. G. J. M. O. Lansink. 2012. Framework for modelling economic impacts of invasive species, applied to pine wood nematode in Europe. PLoS ONE 7:e45505. 23029059 10.1371/journal.pone.0045505 PMC3447758

[eap2319-bib-0080] Strutt, A., J. A. Turner, R. A. Haack, and L. Olson. 2013. Evaluating the impacts of an international phytosanitary standard for wood packaging material: global and United States trade implications. Forest Policy and Economics 27:54–64.

[eap2319-bib-0081] Suckling, D. M., J. M. Kean, L. D. Stringer, C. Cáceres‐Barrios, J. Hendrichs, J. Reyes‐Flores, and B. C. Dominiak. 2016. Eradication of tephritid fruit fly pest populations: outcomes and prospects. Pest Management Science 72:456–465. 25204807 10.1002/ps.3905

[eap2319-bib-0082] Surkov, I. V., A. G. J. M. Oude Lansink, and W. van der Werf. 2009. The optimal amount and allocation of sampling effort for plant health inspection. European Review of Agricultural Economics 36:295–320.

[eap2319-bib-0083] Tobin, P. C., J. M. Kean, D. M. Suckling, D. G. McCullough, D. A. Herms, and L. D. Stringer. 2014. Determinants of successful arthropod eradication programs. Biological Invasions 16:401–414.

[eap2319-bib-0084] Turner, J., L. Bulman, B. Richardson, and J. Moore. 2004. A cost–benefit analysis of forest health and biosecurity research. New Zealand Journal of Forestry Science 34:324–343.

[eap2319-bib-0085] Turner, J. A., and J. Buongiorno. 2004. Estimating price and income elasticities of demand for imports of forest products from panel data. Scandinavian Journal of Forest Research 19:358–373.

[eap2319-bib-0086] Turner, J. A., J. Buongiorno, S. Zhu, and J. P. Prestemon. 2007. Modelling the impact of the exotic forest pest Nectria on the New Zealand forest sector and its major trading partners. Journal of Forestry Science 37:383–411.

[eap2319-bib-0087] Vilà, M., and P. E. Hulme. 2017. Non‐native species, ecosystem services, and human well‐being. Pages 1–14 *in* Impact of biological invasions on ecosystem services. Springer International Publishing, Cham.

[eap2319-bib-0088] Weir, S., and G. Andrews. 2005. Clover root weevil economic impact assessment. Report to Biosecurity New Zealand and Ministry of Agriculture and Forestry. New Zealand Institute of Economic Research (NZIER), Wellington, New Zealand.

[eap2319-bib-0089] White, T. A., and P. J. Gerard. 2006. Modelling the farm scale impacts of clover root weevil herbivory. New Zealand Plant Protection 59:312–316.

[eap2319-bib-0090] Williams, H. P. 2013. Model building in mathematical programming. John Wiley & Sons, Hoboken, New Jersey, USA.

[eap2319-bib-0091] Wittmann, M. E., R. M. Cooke, J. D. Rothlisberger, and D. M. Lodge. 2014. Using structured expert judgment to assess invasive species prevention: Asian carp and the Mississippi Great Lakes hydrologic connection. Environmental Science & Technology 48:2150–2156. 24467555 10.1021/es4043098 PMC3963436

[eap2319-bib-0092] Woodroffe, N. 2010. Analysis of ISPM 15 and its impact on the wood pallet industry. Drake Journal of Agricultural Law 15:199.

[eap2319-bib-0093] Yates, C. M., and T. Rehman. 2002. Development of a partial equilibrium model of the EU agriculture using positivistic mathematical programming. European Association of Agricultural Economists, Zaragoza, Spain.

[eap2319-bib-0094] Yemshanov, D., R. G. Haight, F. H. Koch, B. Lu, R. Venette, R. E. Fournier, and J. J. Turgeon. 2017. Robust surveillance and control of invasive species using a scenario optimization approach. Ecological Economics 133:86–98.

[eap2319-bib-0095] Yemshanov, D., R. G. Haight, F. H. Koch, R. C. Venette, T. Swystun, R. E. Fournier, M. Marcotte, Y. Chen, and J. J. Turgeon. 2019. Optimizing surveillance strategies for early detection of invasive alien species. Ecological Economics 162:87–99.

[eap2319-bib-0096] Young, L. 2002. Determining the discount rate for government projects. Working paper. New Zealand Treasury, Wellington, New Zealand.

