# Supplementary material for: Approaches for estimating benefits and costs of interventions in plant biosecurity across invasion phases
Source: Ecol Appl. 2021 May 6;31(5):e02319. doi: 10.1002/eap.2319 (PMC8365635; doi:10.1002/eap.2319)
Supplement: Supplementary file 1 — Appendix S1 [file EAP-31-e02319-s001.pdf]

Supporting Information. Welsh, M.J., J.A. Turner, R.S. Epanchin-Niell, J.J. Monge, T. Soliman, A.P. Robinson, J.M. Kean, C. Phillips, L.D. Stringer, J. Vereijssen, A.M. Liebhold, T. Kompas, M. Ormsby, and E.G. Brockerhoff. 2021. Approaches for estimating benefits and costs of interventions in plant biosecurity across invasion phases. Ecological Applications.

Table S1. The number of papers using each approach to estimate the costs and benefits of each pest impact type and intervention stage.

	Cost Accounting	Partial Equilibrium	Computable General Equilibrium	Input-output analysis	Empirical Extrapolation	Statistical Modelling	Mathematical Optimisation	Numerical Analysis	Meta-Analysis	Total
Pathway Risk Management	2	2	5	1	0	2	2	0	0	9
Surveillance	6	0	0	0	3	5	8	6	0	16
Eradication and containment	6	0	0	0	1	5	10	8	0	19
Direct Impacts	20	6	6	1	5	10	7	7	1	25
Indirect Impacts	20	7	7	1	4	9	4	5	0	34
Control Costs	14	2	1	0	5	8	7	5	0	41
Total	27	7	7	2	8	13	13	11	1	

Table S2. The number of papers using each approach in combination with each other approach. The diagonal contains the total number of papers using each approach. The bottom two rows summarise how often each approach was used in combination with **at least** one other approach.

	Cost Accounting	Partial Equilibrium	Computable General Equilibrium	Input-output analysis	Empirical Extrapolation	Statistical Modelling	Mathematical Optimisation	Numerical Analysis	Meta-Analysis
Cost Accounting	**27**	1	2	2	5	3	2	2	0
Partial Equilibrium	1	**7**	2	0	0	3	0	2	0
Computable General Equilibrium	2	2	**7**	0	0	1	0	0	0
Input-output analysis	2	0	0	**2**	0	0	0	0	0
Empirical Extrapolation	5	0	0	0	**8**	1	1	1	1
Statistical Modelling	3	3	1	0	1	**13**	6	4	0
Mathematical Optimisation	2	0	0	0	1	6	**13**	8	0
Numerical Analysis	2	2	0	0	1	4	8	**11**	0
Meta-Analysis	0	0	0	0	1	0	0	0	**1**
Number of times approach is used in combination	14	6	4	2	8	13	13	11	1
Percentage of use in combination	51.85%	85.71%	57.14%	100.00%	100.00%	100.00%	100.00%	100.00%	100.00%

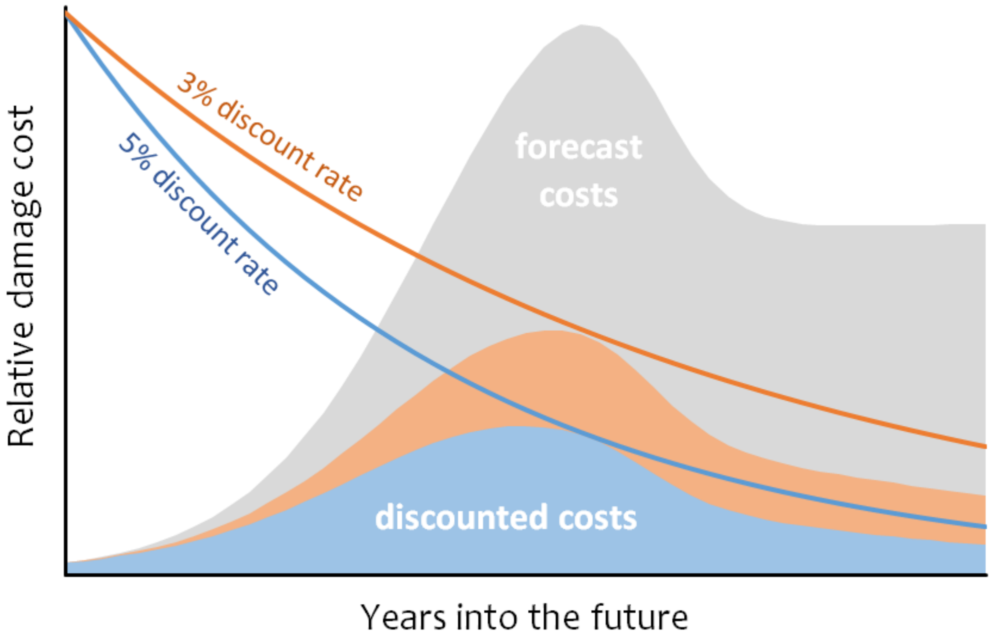

Figure S1. Relationships between discount rate and relative damage costs over time which can affect how investments to mitigate future damages are valued. Relative discounted costs under a 5% and 3% exponential discount rate are indicated by the blue and orange lines, respectively. The grey curve shows a typical damage profile (with no discounting) for an invasive pest with low initial impact that increases over time as the invasion spreads, then decreases as ecosystems adjust and land managers respond. At a 5% exponential discount rate, the damage profile is evaluated as the blue area, with 25% of the forecast costs (grey area) in this example. At a 3% discount rate (orange), this is almost twice as large, with >40% of the forecast costs. This demonstrates how lower discount rates justify greater investment now, to mitigate future damages.

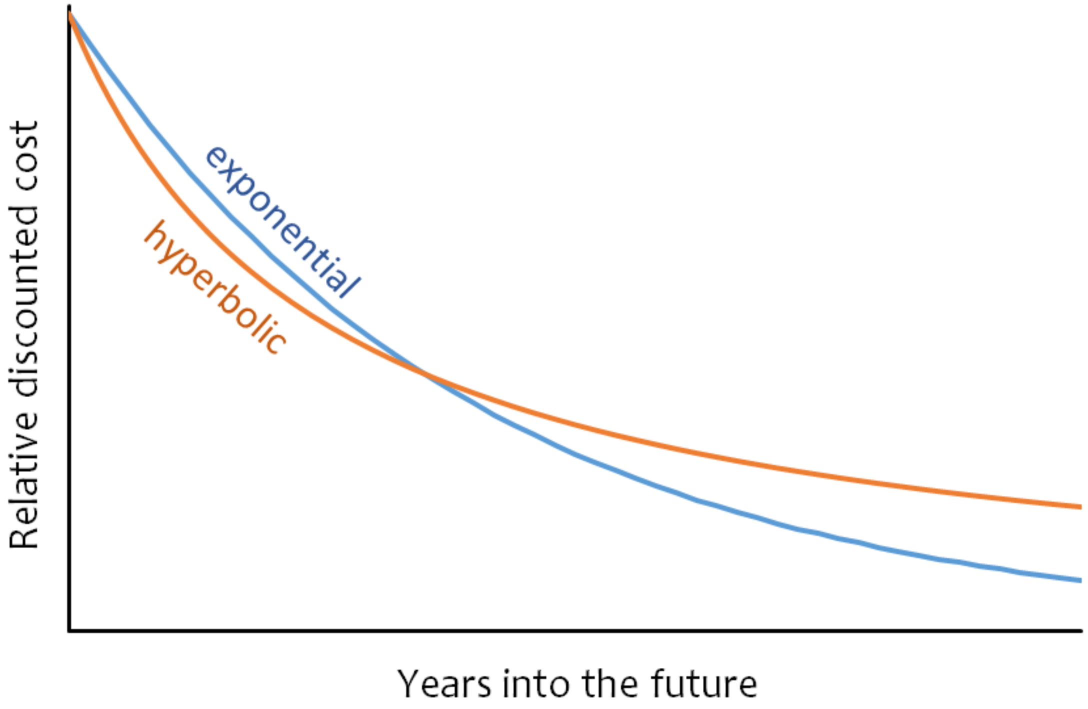

Figure S2. Hyperbolic discounting captures the way that humans instinctively value immediate versus distant rewards. Compared to the temporally consistent exponential method, hyperbolic discounting values short term outcomes more highly than medium term ones (greater initial slope), and treats long term outcomes more equally (longer, flatter tail).
