# Supplementary material for: Approaches for estimating benefits and costs of interventions in plant biosecurity across invasion phases
Source: Ecol Appl. 2021 May 6;31(5):e02319. doi: 10.1002/eap.2319 (PMC8365635; doi:10.1002/eap.2319)
Supplement: Supplementary file 2 — Data S1 [file EAP-31-e02319-s002.zip › MetadataS1.pdf]

Welsh, M.J., J.A. Turner, R.S. Epanchin-Niell, J.J. Monge, T. Soliman, A.P. Robinson, J.M. Kean, C. Phillips, L.D. Stringer, J. Vereijssen, A.M. Liebhold, T. Kompas, M. Ormsby, and E.G. Brockerhoff. 2021. Approaches for estimating benefits and costs of interventions in plant biosecurity across invasion phases. Ecological Applications.

Data S1

Spreadsheet detailing the papers reviewed for this manuscript.

Authors

Melissa J. Welsh
Scion (NZ Forest Research Institute)
P.O. Box 29237, Christchurch 8540, New Zealand
melissa@welsh.co.nz

James A. Turner
AgResearch, Ruakura
10 Bisley Rd, Hamilton, New Zealand
james.turner@agresearch.co.nz

Eckehard G. Brockerhoff
Swiss Federal Research Institute WSL, Zürcherstrasse
111, 8903 Birmensdorf, Switzerland
eckehard.brockerhoff@gmail.com

Andrew Robinson
Centre of Excellence for Biosecurity Risk Analysis
School of BioSciences, University of Melbourne, Australia
apro@unimelb.edu.au

File list (files found within DataS1.zip)

`PaperReview.csv`

Description

`PaperReview.csv` – A spreadsheet containing a list of the papers reviewed in detail for this manuscript. This spreadsheet contains a description of the methods used in each paper, which sectors they apply to, what types of impacts are discussed and what stages of the biosecurity spectrum are covered. The time frame covered by the paper and any notable shortcomings are also listed.
